# Monitoring of Pentoxifylline Thermal Behavior by Novel Simultaneous Laboratory Small and Wide X-Ray Scattering (SWAXS) and Differential Scanning Calorimetry (DSC)

**DOI:** 10.1371/journal.pone.0159840

**Published:** 2016-07-28

**Authors:** Aden Hodzic, Manfred Kriechbaum, Simone Schrank, Franz Reiter

**Affiliations:** 1 Central European Research Infrastructure Consortium (CERIC-ERIC), Trieste, Italy; 2 Institute of Inorganic Chemistry, Graz University of Technology, Graz, Austria; 3 Research Center Pharmaceutical Engineering GmbH, Graz, Austria; 4 GL-Pharma GmbH, Lannach, Austria; 5 Institute of Pharmaceutical Sciences, University of Graz, Graz, Austria; University of Colorado Anschutz Medical Campus, UNITED STATES

## Abstract

The thermal and structural evolutions associated to active pharmaceutical ingredient (API) purity are monitored using a laboratory instrument (S3-MicroCaliX) allowing simultaneous time-resolved X-ray scattering at both wide and small angles (SWAXS) as a function of temperature. This is performed simultaneously with differential scanning calorimetric (DSC) that is carried out in the same apparatus at scanning rate of 2 K/min on the same sample in the range from 20° to 200°C. We have studied simultaneous thermal and structural properties of pentoxifylline, as an active pharmaceutical ingredient (API), for its purity quality control. We have found a satisfying API purity, due to obtained melting temperature and enthalpy values, which are in a well agreement with literature. We have also found that the combination of these techniques allows the thermal monitoring of scanning rates of 2 K/min, continuously without the need for static thermal equilibration, particularly for X-ray spectra. Hence, DSC and SWAXS allowing better identification of the structural thermal events recorded by following of the phase transitions simultaneously. This interpretation is much better possible when X-ray scattering at small and wide angles is coupled with DSC from the same sample. Hence, as a laboratory tool, the method presents a reproducible thermal and crystallographic API purity quality control of non-complex samples, as crucial information for pharmaceutical technology.

## Introduction

Analytical quality control of APIs is a key to follow quality of pharmaceutical processes and products. A strict testing to ensure the absence of destructive impurities is highly relevant for any further pharmaceutical procedure. Also, recognizing of API polymorphic state and structural identification before and after pharmaceutical procedure is an important part of a product quality control. Crucial API impurities can be detected alone with phase transition and thermal behaviors. Technique used for such thermal analytics are differential scanning calorimetry (DSC), differential thermal analysis (DTA), thermomechanical analysis (TMA, thermogravimetric analysis (TGA), [1]. Here we can mainly follow material phase transitions, but no possibility of API crystalline structural characterization.

Standard methods for structural characterization i.e. API polymorphism are based on X-ray diffraction and scattering. Classical X-ray method as wide angle X-ray scattering (WAXS), yields an extended range of Bragg peaks [2] (“finger-prints”) as material identification and material polymorphic state information, but does not allow any analytics of larger Nano-structural information contained in the small angle X-ray scattering (SAXS) region (2Ѳ < 6°). SAXS analytics provides information about inhomogeneity’s in the nm-range and thus enables an investigation of shape and size of macromolecules, characteristic distances of partially ordered materials and pore sizes, for instance. SAXS analytics in pharmaceutical powders may serve a determination of Nano -scopical total inner surface, correlation length and fractal dimension within Nano-domains. Anyway, combination of the SAXS and WAXS leads to a more powerful analytical combined tool SWAXS. Usefulness of SAXS and WAXS has been well demonstrated by numerous applications in bioscience, material and polymer science [3, 4, 5]. In addition to static studies, SWAXS has been used to investigate dynamic systems, in which morphological responses to chemical reaction, mechanical deformation with temperature changes can be observed.

On the other hand, DSC has been also used for various applications in the pharmaceutical industry. These applications include studying polymorphism of APIs, evaluating the stability of storage conditions of drug products/APIs/raw materials [6], the quantization of pharmaceutical crystal forms [7] and for the purity determination of crystalline organic compounds [8, 9]. DSC is a thermo analytical technique in which the difference in the amount of heat required to increase the temperature of a sample is measured relative to a reference as a function of temperature. Both the sample and reference are maintained at nearly the same temperature throughout an experiment. When the sample undergoes a physical transformation the heat flux is either increased or decreased, depending on whether the process is exothermic or endothermic. The endothermic melting of a solid will increase the heat flow into the sample, whereas the exothermic crystallization will reduce the heat required to raise the sample temperature. By observing the difference in heat flow between the sample and reference, the amount of heat absorbed or released during the transitions indicates phase changes of the material. This makes DSC a relevant asset in sample purity, by investigating phase transition events. The advantage of purity analysis by DSC is that it does not require a corresponding reference standard but a minimal sample amount and short total analysis. There again, the disadvantage is the lack to clearly distinguish between a crystalline and amorphous thermal transition. Thus, a coupling with an X-ray scattering method allows resolving this issue.

Although both methods, SWAXS and DSC, provide relevant information for raw material and product analysis, the combination of both methods into one instrument, and the simultaneous analysis of a single sample provide more analytical advantages. In general, simultaneous experiments by different methods on a single sample offer great advantages over separate (sequential) experiments avoiding artifacts. Hence, not only time is saved but also errors due to differences in, for instance, sample environment, thermal history, age, concentration, temperature and sample preparation can be eliminated completely. The possibility to resolve ambiguities in the understanding of phase-transition mechanisms by allowing accurate determination of the order of the events occurrence by the different techniques is even more important. Combining these two methods, SWAXS and DSC, crystallinity, phase and polymorph identification is followed simultaneously with scattering information, which allows better structural thermal analytics.

Nevertheless, there appears to be no previous introduction of a combined SWAXS/DSC method integrated simultaneous in a single laboratory instrument. However, such technique was previously demonstrated at synchrotron X-ray sources [10], but not everyone has easy access to such source at any time. Also the repeatability and time flexible experimental planning are not that much possible at a synchrotron beam-line due to access limitations. This was the motivation for such laboratory instrument and hence we present the first commercially available laboratory SWAXS/DSC system called S3-MICROcaliX (Hecus X-Ray Systems) and its application in the API solid-state purity quality control.

Furthermore, most commercially available DSC instruments are not suited to performing combined DSC and X-ray scattering experiments. For accurate DSC analysis, the sample has to be enclosed in an environment shielded from temperature fluctuations; because of the absorption of X-rays by the necessary windows, this has an adverse effect on the collection of the X-ray data. Thus, a modified type of DSC cell has been developed that can overcome this problem and can be utilized for the above-described experiments. First results of this innovative system applied in pharmaceutical quality control applications for non-complex samples are presented in the following.

## Materials and Methods

### Sample Preparation and Measuring

**Fig 1:**
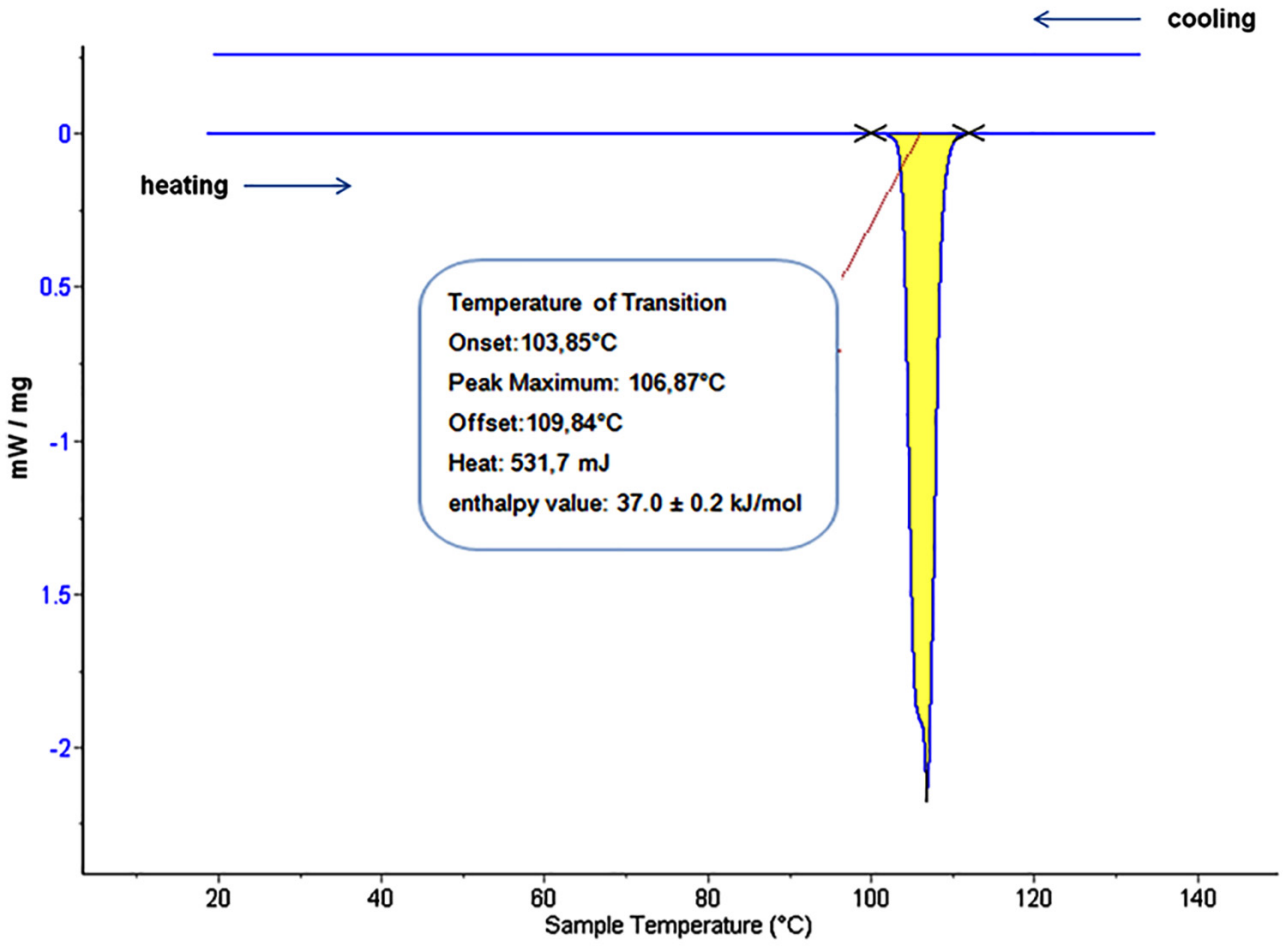
DSC spectra of pentoxifylline: Heating scan with a base line subtraction and slope correction displaying melting transition. Cooling scan with a base line subtraction displays a stabile amorphous state.

We have used pentoxifylline as an API for purity analytics and SWAXS/DSC method application. Pentoxifylline (3,7-dimethyl-1-(5-oxohexyl)-3,7-dihydro-1Hpurine-2,6-dione) is a synthetic dimethyl xanthine derivative structurally related to theophylline and caffeine (Fig 1). It is a nonselective phosphodiesterase inhibitor and allows the erythrocyte membrane to maintain its integrity as well as resistance to deformity. It is used for the treatment of peripheral vascular diseases, management of cerebrovascular insufficiency, sickle cell disease, and diabetic neuropathy [11, 12, 13].

Pentoxifylline samples were obtained from the GL Pharma (Lannach, Austria). For measurements, the samples (4 mg) were filled into quartz capillaries of 1.5 mm inner diameter and placed in the measuring chamber of the S3-MICROcaliX. Time resolved SWAXS spectra and DSC-signals were recorded at a scan-rate of 2 K/min (found as a propriety scan rate for the simultaneous technique), and at an X-ray exposure time of 60 s for each frame, applying a beam diameter size of 200 μm.

### SWAXS/DSC

The S3-MICROcaliX combines proprietary technologies in X-ray optics and calorimetry and presents a combination of SWAXS and DSC methods, implanted in one single instrument applicable for such simultaneous analytics.

SWAXS (SAXS and WAXS) system contains a high-flux laboratory small- and-side angle X-ray scattering camera S3-Micro (Hecus X-Ray Systems, Graz Austria) equipped with a high-brilliance micro-beam delivery system, operating at a low power of 50W (50kV and 1 mA), with point-focus optics (FOX3D, Xenocs, Grenoble, France) and with 1D or 2D -detection system combined with automated data acquisition software. The X-ray wavelength λ was 1.54 Å and the SAXS and WAXS -curves (scattering intensities as a function of the scattering angle 2θ) were recorded with two independent 1D-detectors (PSD-50, Hecus X-ray Systems, Graz, Austria) in the angular range 0.06° < 2 θ < 8° and 17° < 2 θ < 27°, respectively. The q-scale is related to the scattering angle 2θ by q = 4π*sin θ/λ and represents the magnitude of the reciprocal scattering vector. The calibration of the q-scale, i.e. the calibration of the detector pixels to the respective scattering angle, was done by measuring silver-behenate with a defined lamellar spacing of 5.838 nm in the SAXS range and by p-bromo-benzoic-acid in the WAXS range, respectively.

In addition the integrated DSC system (Setaram, Caluire, France) allows performing temperature scanning experiments with rates up to 120°/h, a dynamic calorimeter range of ± 190 mW and a sensitivity of 0.02 μW. We used a heating and cooling scan rate of 2°C/min with an exposure time of 60 seconds for every X-ray spectrum (1 frame/2°C). The Microcalix DSC apparatus was calibrated with a 0.999999 mol fraction purity indium sample. The repeatability of the melting temperature was 0.5 K. Data evaluation of the DSC measurements was done by CALISTO Thermal Analysis Software (SETARAM, Caluire, France). All presented DSC curves were background-slope corrected and the thermal parameters obtained were calculated by this software package. As a reference an empty capillary was taken. Calibration of the thermal signal was done by Indium metal.

Concluding, such instrumentation allows the measurements of nanostructural and calorimetric changes, phase transitions, structural changes, reactions in solid powders, pastes and liquid samples simultaneously in one single experiment.

## Results and Discussion

We report about SWAXS/DSC simultaneous laboratory technique application for an API quality control, probing the API structural (by SWAXS) and the melting (by DSC) behavior whose combination provides information about material quality and purity.

In general, DSC is a technique in which the heat flux (thermal power) to (or from) a sample is measured versus time or temperature while the temperature of the sample is changing in a controlled way. The difference of heat flux between a crucible containing the sample and a reference crucible (empty or not) is measured and displayed. The pharmaceutical analytics often use the DSC curve of quench-cooled API´s to illustrate the sequence of devitrification, crystallization and melting. Therefore, in order to evaluate the API-purity of pentoxifylline, we have characterized its thermal and nanostructure behavior by simultaneous SWAXS/DSC.

**Fig 2:**
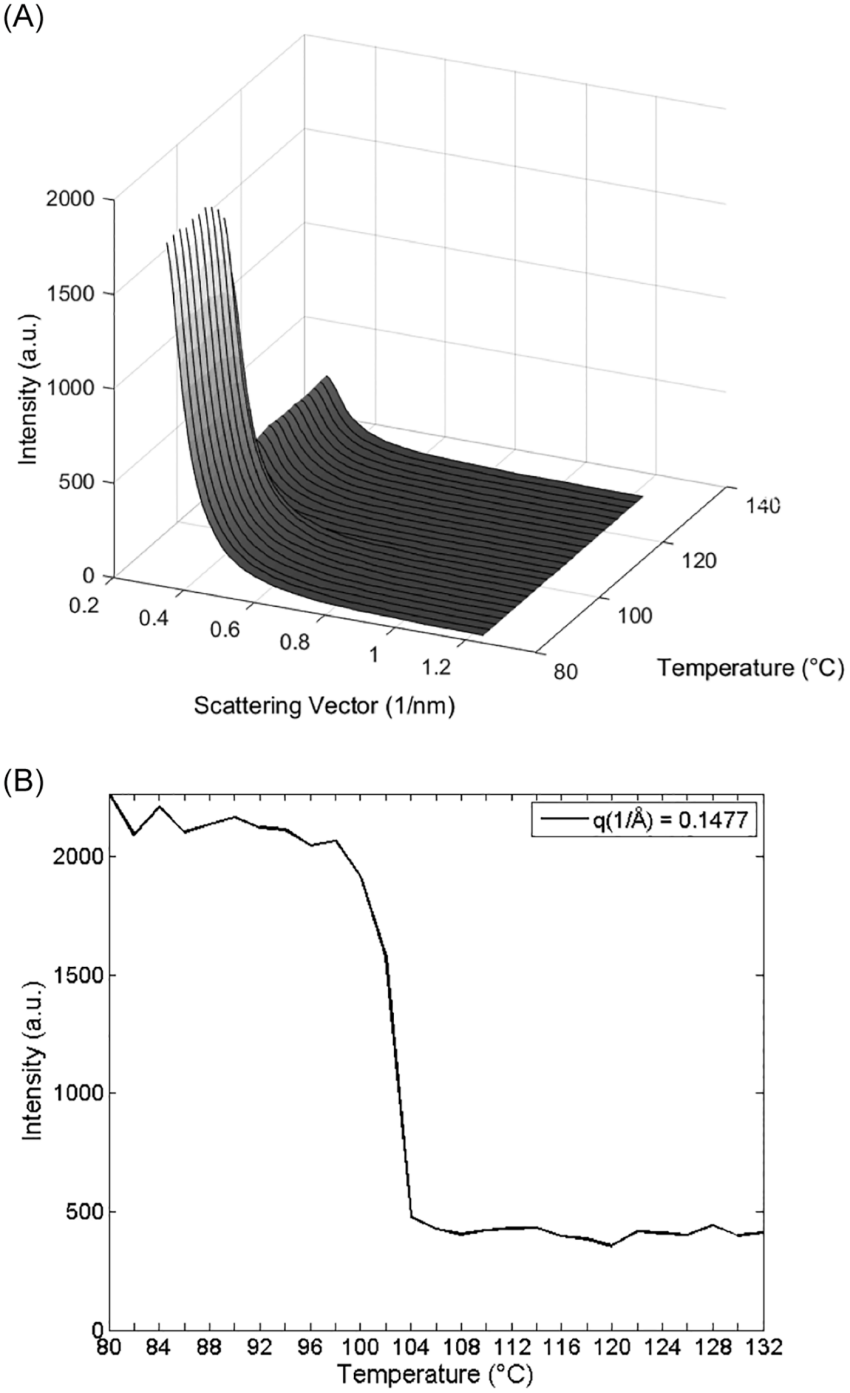
SAXS heating scans spectra of pentoxifylline in the temperature range of 80 to 140°C: a SAXS heating scan, b) SAXS scattered intensity versus temperature. (SAXS exposure time one minute per frame, which corresponds to two°C per frame).

**Fig 3:**
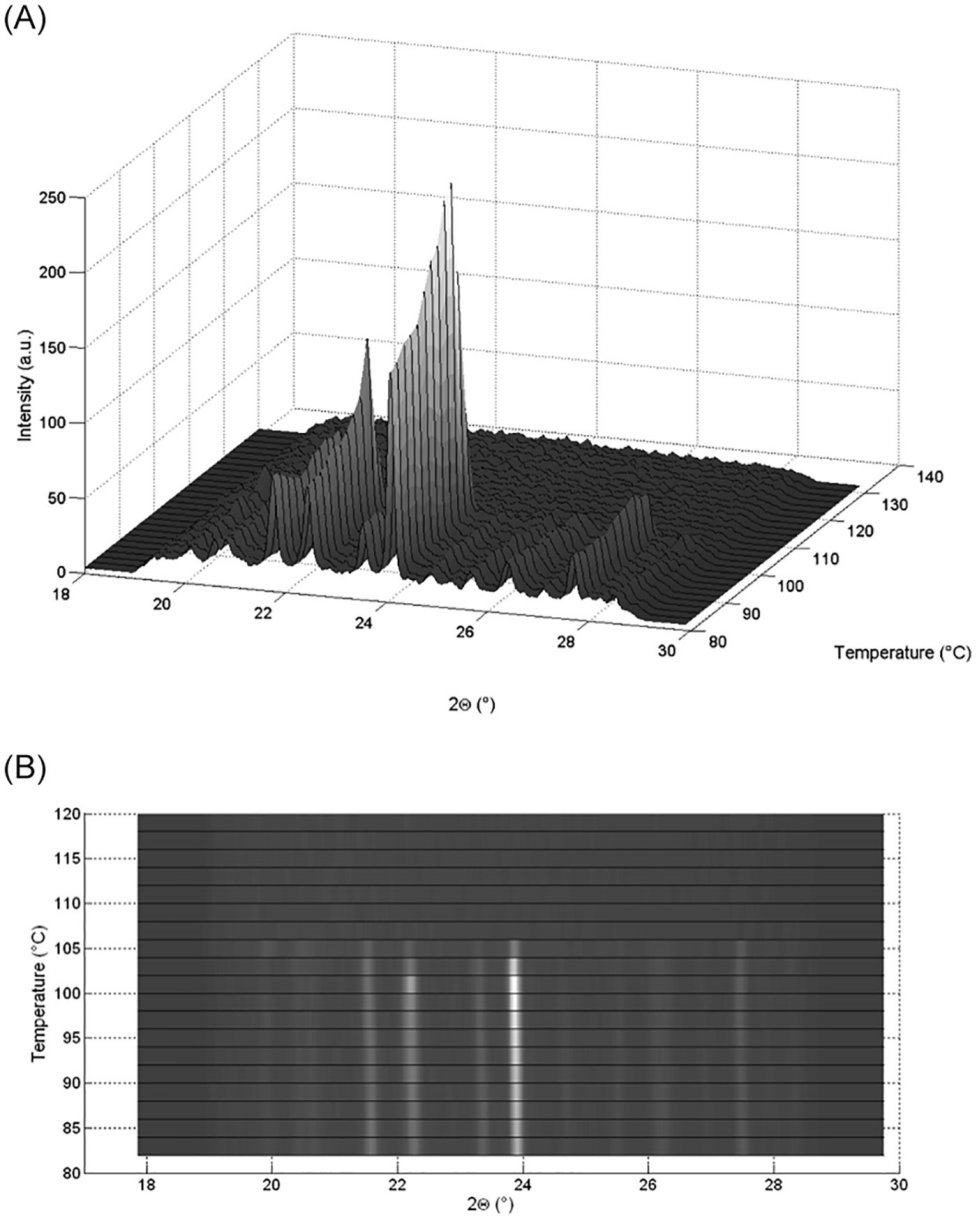
a) WAXS heating scan in the three-dimensional plot, b) WAXS heating scans in a two dimensional contour plot. (SAXS exposure time one minute per frame, which corresponds to two°C per frame).

Fig 1 is a DSC curve for the heating and the subsequent cooling scanning experimental procedure. DSC-signals have been recorded simultaneously with scattering patterns presented as a three-dimensional plot of l (scattered intensity expressed in arbitrary units (a.u.)) versus q (scattering vector) and versus T (temperature) by SAXS in Fig 2a and versus 2 Ѳ (scattering angle) and versus T by WAXS in Fig 3a and S2 Fig, respectively. In the heating scan, DSC displays the main thermal event change shown by the endothermic melting peak maximum at the temperature of 107.01°C ± 0.5°C (Fig 1). Repetition experiments show a well melting temperature reproducibility (0.5°C) and a good agreement with the reported temperature value [14], which indicates a high purity of the analyzed API. It also shows a thermal accuracy of the presented new instrumentation. The crystal melting process was indicated by DSC first with an onset temperature of 103.85°C and after further going through the maximum of melting peak (107.1°C), the process ended with a temperature offset of 109.84°C (Fig 1). This relative narrow range between onset and offset melting temperatures with the sharp endothermic peak again indicates a presence of an API without substantial impurities. Furthermore, the integral over the narrow peak area (Fig 1) results a heat value of 531.7 ± 0.2 (mJ). This value divided by the weight of the measured sample (4mg) and then multiplied by the molecular weight of pentoxifylline (278.31 g/mol) yields an enthalpy value of 37.0 ± 0.2 kJ/mol for the API melting transition. Compared to the literature value, which is 36.6 kJ/mol [15], it is in a good experimental agreement and proves the purity of the API.

**Fig 4:**
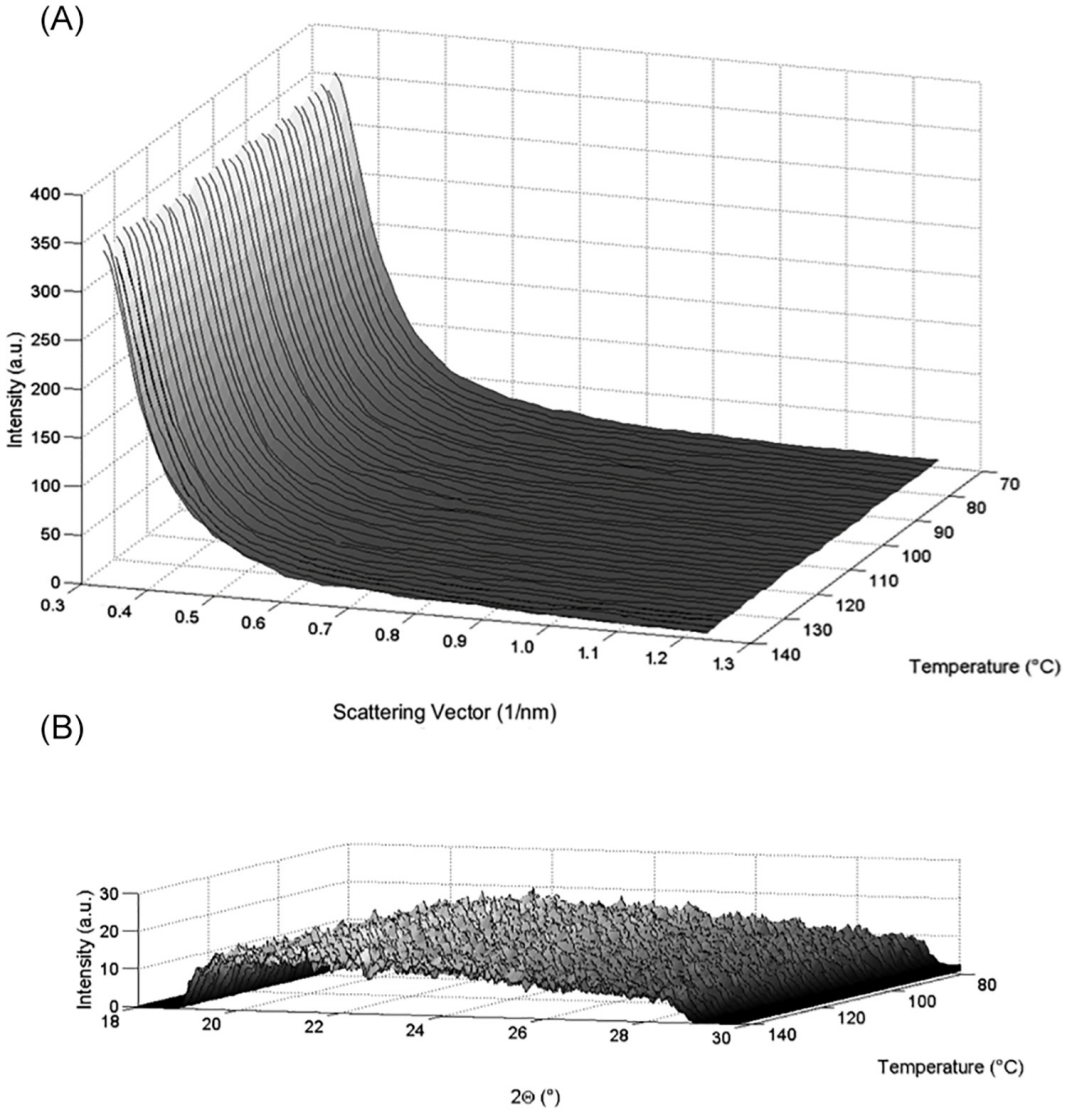
SWAXS cooling scans spectra of pentoxifylline in the temperature range of 140 to 80°C: a) SAXS cooling scan, b) WAXS cooling scan in the three dimensional plot.

In order to check the thermodynamic correlation of the SAXS patterns and the DSC curve, we have zoomed the scattering power at low q values (Fig 2). The melting behavior is observed in the changes of the time-resolved SAXS patterns in the diffuse scattering (no Bragg peaks in the SAXS region) intensities. Scattering intensities towards lower (~0) q values (SAXS region in the Fig 2a and S1 Fig) decrease as individual crystals melt. The SAXS scattering intensity on the onset temperature begins to decrease until it reaches a minimum (offset temperature), which remains constant in the further heating procedure in the displayed temperature range (see Fig 2). Hence, it is possible to follow simultaneously nanostructural and thermodynamic fluctuations of crystalline behavior even before melting, especially at small q values (here q < 0.4 nm^-1^). It marks SAXS as a method sensitive to such thermal changes (Fig 4a) close to the zero angle region of scattering, which cannot be determined by classical X-ray-diffraction (XRD) due to its angular resolution lacking the SAXS range. The scattering power can also be parameterized as an integral over the zeroth, first or second (“invariant”) moment of the scattering curve (for more information see [16, 17]), which reveals the same trend (data not shown) as obtained here by SAXS curves intensities behavior (Fig 2b). The onset on the DSC is at 104°C, the peak at 107°C and the offset at 108.5°. The thermal signal (at the heating rate of 2°C/min) of the DSC is measured continuously whereas the exposure time of SAXS is one minute (2°C per spectrum) in order to get a SAXS-curve with reasonable statistics. Thus, one frame of the SAXS data contains also the changes which happen during the next 2°C. Therefore the slight discrepancy in the synchroisation of the data with both methods. The drastic decrease of the SAXS-intensity (102°C) occurs then already with the onset of the melting (104°C).

Furthermore, the WAXS patterns resulting from the crystalline form before—and amorphous form after melting—are shown in Fig 3a and 3b with the respective DSC-signal (Fig 1). The WAXS 2D plot (Fig 3b) shows that the crystals are transforming throughout the heating process and the peak positions at 2θ move to lower angles as the crystal lattice increases with increasing temperature. The peak movement is about ~ 0.4° 2θ value for all visible Bragg peaks (Fig 3b), which indicates a slight thermal undulation and expansion of the crystal lattice before melting. Additionally, the WAXS data (Fig 3a and S2 Fig) with the scattering intensity of the highest Bragg-peak at the 23.5° (2θ), increase continuously as the lattice expands and then melts. The melting procedure by WAXS is also in agreement with the DSC data and shows the melting temperature about ~107°C with an acceptable error range of 0.5°C.

In addition, scattered intensity contains information about the degree of crystallinity, since more ordered crystals exhibit higher scattered intensity. That is why such intensity decreases after melting when the sample transforms into an amorphous state (Fig 4b). The temporal SWAXS/DSC signal synchronization of the melting temperature event is well in mutual agreement due to the fact that the result is from a simultaneous experiment. The SWAXS/DSC peak-to-peak correlation is excellent because the timing markers allowed such correlation in the simultaneous experiment. The correlation is most visible in the heating scans, due to the thermal events by melting of the API crystal lattice to an amorphous state.

The cooling procedure under the same scanning rate (2°C/min and each 60 seconds record of SWAXS spectra) illustrates an irreversibility of melting by formation of a constant amorphous structure. However, thermal transitions or fluctuations by cooling are not reflected in any changes of the scattering intensity by the SAXS signal (Fig 4a), which indicates a stable amorphous state of the API in the displayed temperature range. Also quality parameters obtained in the diffuse SAXS range, such as Porod exponent, invariant and inner surface [14, 15], show no significant differences between the first and the last frame of the cooling scan (Fig 4a) by the amorphous state (therefore this data are not shown). Thus, these data prove the stable API amorphous state after melting over the entire cooling temperature range.

## Conclusion

In general, coupling of time-resolved laboratory X-ray diffraction both, at small and wide angles with differential scanning calorimetry is a new laboratory tool that allows simultaneous characterization of thermal and structural properties of the same sample, as shown in the reported example of pentoxifylline with respect to quality/purity control. Differential scanning calorimetry (DSC) alone allows the characterization of the changes of a state and more generally the reversibility of the phenomena involved in the phase transitions between condensed states. However, this technique does not inform about exact structure evidence before and after the phase transition recorded. The characterization of these transitions is often complicated by the existence of a polymorphism (capability of a substance to be crystallized under various different forms). Thus, a simultaneous combination with SWAXS allows in addition structural information about a sample for any step of such thermal transition. Hence, the use of this instrument is obviously meant to link simultaneously thermodynamic changes accurately with structural changes. The advantage is not only an economical, but also a qualitative one, since errors and uncertainties that previously could originate from different experimental sample environment, history, preparation etc. are eliminated. The DSC/SWAXS coupling has been already proposed in experiments at synchrotron X-ray sources [18, 19]. Since the accessibility to synchrotrons is limited, the S3Microcalix allows for the first time a DSC coupling simultaneously with X-ray scattering (SWAXS) in a laboratory environment, and hence presents a useful tool also for quality control and formulations studies in the pharmaceutical industry. The combination of both complementary methods opens new perspectives for understanding material properties, in particular for pharmaceutical technology.

## Supporting Information

S1 Fig This is the S1 Fig Title. SAXS heating scans spectra of pentoxifylline in the temperature range of 80 to 140°C: SAXS heating scan.
(TIF)

S2 Fig This is the S2 Fig Title. WAXS heating scan in the two dimensional plot.
(TIF)
