# Supplementary figures and images for: Monitoring of Pentoxifylline Thermal Behavior by Novel Simultaneous Laboratory Small and Wide X-Ray Scattering (SWAXS) and Differential Scanning Calorimetry (DSC)
Source: PLoS One. 2016 Jul 28;11(7):e0159840. doi: 10.1371/journal.pone.0159840 (PMC4965141; doi:10.1371/journal.pone.0159840)

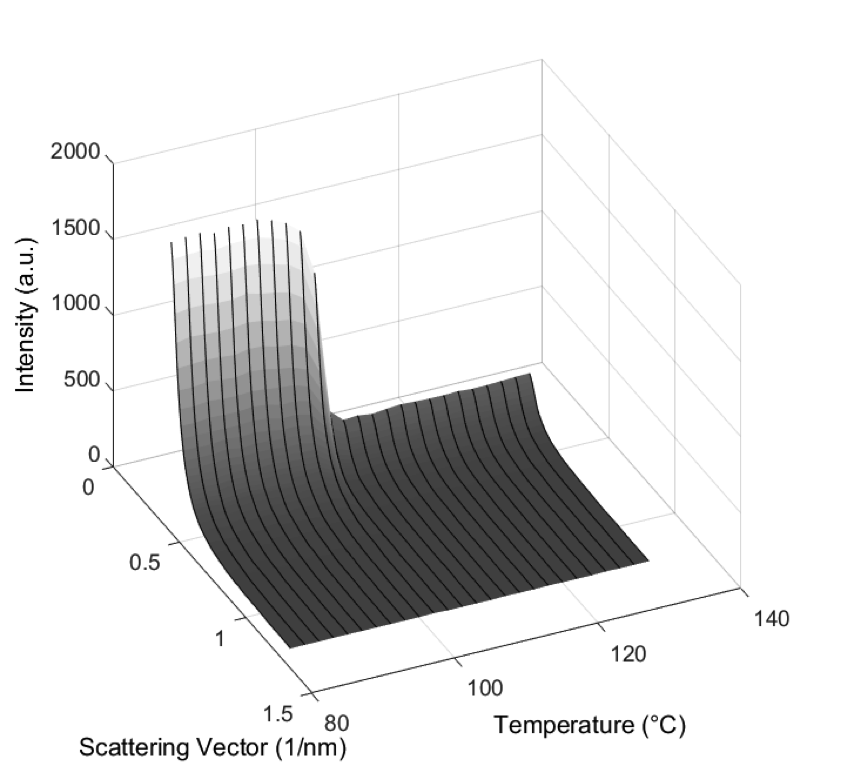

Supplement: S1 Fig — SAXS heating scans spectra of pentoxifylline in the temperature range of 80 to 140°C: SAXS heating scan. (TIF) [file pone.0159840.s001.tif]

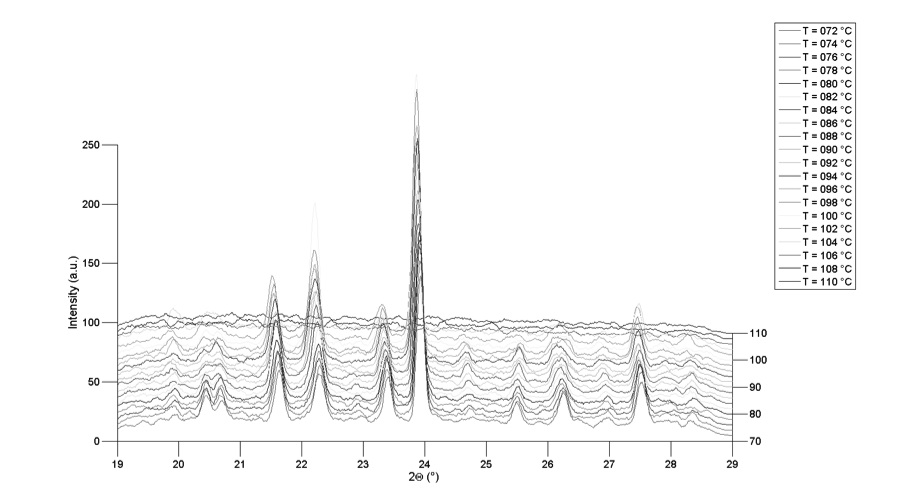

Supplement: S2 Fig — WAXS heating scan in the two dimensional plot. (TIF) [file pone.0159840.s002.tif]
